# Evaluating the Impacts of Procedural and Patient-Specific Factors on the Outcomes of Transcatheter Aortic Valve Implantation (TAVI)

**DOI:** 10.3390/medicina61010094

**Published:** 2025-01-09

**Authors:** Abilkhair Kurmanaliyev, Rima Braukylienė, Ali Aldujeli, Rassul Zhumagaliyev, Serik Aitaliyev, Ramunas Unikas

**Affiliations:** 1 Department of Cardiology, Hospital of Lithuanian University of Health Sciences Kauno Klinikos, Lithuanian University of Health Sciences, 2 Eivenių Str., LT-50009 Kaunas, Lithuania; abilxaer1@gmail.com (A.K.); rima.braukyliene@lsmu.lt (R.B.); ali.aldujeli@universityofgalway.ie (A.A.); ramunas.unikas@yahoo.com (R.U.); 2 CORRIB Research Centre for Advanced Imaging and Core Laboratory, University of Galway, 1 University Road Str., H91 TK33 Galway, Ireland; 3 Department of Cardiac, Thoracic and Vascular Surgery, Hospital of Lithuanian University of Health Sciences Kauno Klinikos, Lithuanian University of Health Sciences, 2 Eivenių Str., LT-50009 Kaunas, Lithuania; rasszhum0425@kmu.lt; 4 Faculty of Medicine and Health Care, Al-Farabi Kazakh National University, 71 Al-Farabi Ave., 050040 Almaty, Kazakhstan

**Keywords:** aortic stenosis, transcatheter aortic valve implantation, outcomes, fluoroscopy, survival

## Abstract

*Background*: Transcatheter aortic valve implantation (TAVI) has emerged as a pivotal intervention for managing severe aortic stenosis in high-risk surgical patients. *Objective*: This study aimed to evaluate the impacts of procedural factors and patient characteristics on TAVI outcomes, with a focus on survival rates, cardiac mortality, and associated complications. *Methods*: A retrospective, single-center study involving 224 patients who underwent TAVI at the Lithuanian University of Health Sciences from September 2021 to April 2023 was conducted. Data encompassing demographic characteristics, medical history, procedural specifics, and follow-up outcomes were analyzed. Survival and adverse events were assessed at 30 days, 6 months, and 12 months post-TAVI. *Results:* The study included 224 patients. The mean age in the non-death group was 80 ± 6.17 years (range, 49–91), while that in the cardiac death group was 81.5 ± 6.14 years (range, 70–94; *p* = 0.079). Males accounted for 37.7% of the non-death group and 50% of the cardiac death group (*p* = 0.304). Statistical analyses identified factors significantly associated with mortality and complications. The overall survival rate was 88.8%, with cardiac-related mortality observed in 8% of patients. Increased fluoroscopy time (*p* < 0.001), a higher contrast volume (*p* = 0.005), and less improvement in aortic valve velocity post-TAVI (*p* = 0.031) were significantly associated with cardiac mortality. Advanced age and a reduced left ventricular ejection fraction (<50%) were prominent predictors of adverse outcomes. Patients with non-coronary cusp calcification exhibited lower cardiac mortality (*p* = 0.005), while mitral valve regurgitation was linked to poorer outcomes (*p* = 0.015). Logistic regression analysis underscored the incremental risks posed by procedural complexities and comorbidities. *Conclusions*: Procedural factors such as fluoroscopy duration and contrast volume, along with patient-specific attributes including age, left ventricular function, and valve calcification patterns, critically influence TAVI outcomes. These findings emphasize the need for tailored procedural strategies and patient management protocols to mitigate risks and enhance the efficacy of TAVI interventions.

## 1. Introduction

Cardiovascular diseases (CVDs) continue to be the leading cause of death and disability worldwide, despite significant advancements in their diagnosis and treatment. Aortic stenosis (AS)—a progressive narrowing of the aortic valve opening—is one of the most common and severe forms of valvular heart disease, affecting older adults in particular. Clinically, AS often presents with symptoms such as exertional dyspnea, chest pain, and syncope and, if left untreated, can lead to heart failure and death. Diagnosis typically involves echocardiography, which confirms the severity of valve narrowing and its hemodynamic impact [1].

Transcatheter aortic valve implantation (TAVI) has revolutionized the treatment of severe symptomatic AS, offering a less-invasive alternative to surgical aortic valve replacement (SAVR). The procedure involves the percutaneous delivery of a bioprosthetic valve to replace the diseased aortic valve, typically under fluoroscopic and echocardiographic guidance. TAVI has significantly reduced recovery times compared to SAVR and has expanded treatment options to high-risk patient populations previously deemed unsuitable for surgery due to age, comorbidities, or frailty [2,3].

Despite its transformative impact, there remains a critical need to understand the long-term outcomes and risk factors associated with TAVI. A detailed evaluation of factors such as mortality, survival rates, and cardiac-specific mortality is essential for refining patient selection criteria and optimizing management protocols. Key determinants of post-TAVI outcomes include pre-existing comorbidities, procedural complexities, and post-operative care strategies, emphasizing the need for comprehensive decision making in this context [4,5,6].

Contemporary studies have highlighted the evolution of TAVI techniques, supported by advancements in technology and operator expertise, resulting in improved survival and reduced complications over time. However, further research is needed to optimize procedural strategies and enhance patient care [7,8].

This study aims to analyze a range of factors influencing TAVI outcomes, including patient demographics (age, gender), comorbidities (diabetes, hypertension), and broader cardiovascular history. Laboratory and instrumental parameters augment this analysis, providing a comprehensive understanding of the determinants impacting mortality, survival, and cardiac mortality. These insights contribute to the ongoing discourse on improving TAVI procedures and patient outcomes in the treatment of severe aortic stenosis. [9,10].

## 2. Methods

This retrospective, single-center study was conducted in the cardiology department of the Lithuanian University of Health Sciences Kaunas Clinics from 1 September 2021 to 1 April 2023. The aim was to evaluate the impact of procedural factors on the outcomes of transcatheter aortic valve implantation (TAVI) among 230 individuals diagnosed with severe aortic stenosis. The sample included 224 patients with normal aortic anatomy without aneurysms, normal levels of myocardial injury markers (Troponin I) in the blood, and normal heart structure without atrial septal defects as determined by echocardiography. All patients had severe aortic stenosis and were categorized as high or very high surgical risk, according to the latest recommendations of the European Society of Cardiology [11].

Excluded from the study were lactating and pregnant women; patients suffering from myocarditis or restrictive pericarditis; those requiring surgery for other significant valvular diseases; those with severe calcification of the abdominal and femoral arteries; and those with all types of aortic aneurysms. Six patients were excluded: four who refused to participate; one with severe calcification of the aorta, abdominal, and femoral arteries; and one who was excluded due to access via the subclavian artery.

TAVI procedures were performed based on the decision of the “Heart Team”, which included specialists such as cardiac surgeons, interventional cardiologists, diagnostic specialists, intensivists, and radiologists. Over a one-year follow-up period, comprehensive data were collected, including demographic information, medical history, and post-operative outcomes such as survival and hospital readmission rates. Data collection was completed in April 2024 through outpatient visits or, alternatively, via telephone contact with patients or their physicians.

Clinical, laboratory, and instrumental data were collected retrospectively. This included patient age, gender, comorbidities, history of cardiovascular diseases, and specific procedural variables such as fluoroscopy time and contrast volume, as well as the evaluated change in AoV velocity after TAVI. The study protocol was reviewed and approved by the Institutional Review Board of the Lithuanian University of Health Sciences. All procedures followed were in accordance with ethical standards.

The study received approval from the local ethics committee (BE-2-101), and all participants provided written informed consent, in accordance with established ethical standards.

### 2.1. Echocardiography

All transthoracic echocardiograms were obtained and analyzed by board-certified echocardiographers, according to the standard protocol recommended by the ASE. Baseline echocardiograms were obtained before TAVI and 12–48 h after TAVI. Transthoracic echocardiography was performed using an echocardiograph (Philips North America, Andover, MA, USA).

### 2.2. Computed Tomography

All patients underwent contrast-enhanced computed tomography (CT) of the heart, aorta, and femoral arteries. CT scans were performed at Kauno Klinikos using a 640-slice scanner with simultaneous 0.5 mm slices obtained in 0.275 s per full rotation (Aquilion GENESIS; Canon Medical Systems USA, Inc., Tustin, CA, USA). Patients received 70–90 mL of the contrast agent Iomeron 400 (Patheon Italia, Ferentino, Italy). After CT scanning, the obtained data were processed using the 3Mensio Structural Heart and Vascular software (version 5.1; Pie Medical Imaging, Maastricht, The Netherlands)

### 2.3. Description of the Device

Several types of valves were implanted in patients, including balloon-expandable valves (BEVs) and self-expandable valves (SEVs). The BEVs were Myval (Meril Life Sciences Pvt. Ltd., Mumbai, India) valves in 20 mm, 21.5 mm, 23 mm, 24.5 mm, 26 mm, 27.5 mm, 29 mm, 30.5 mm, and 32 mm sizes; the SEVs included CoreValve/Evolut R/Evolut Pro (Medtronic, Minneapolis, MN, USA) in 23 mm, 26 mm, 29 mm, and 34 mm sizes as well as ACURATE neo2 (Boston Scientific Corp., Marlborough, MA, USA) in the sizes S-23 mm, M-25 mm, and L-27 mm. All of the above-mentioned valves were implanted during the TAVI procedures.

### 2.4. Definitions

Patients were considered to be at high surgical risk when there was consensus that valve replacement surgery could be associated with excessive morbidity or mortality, as confirmed by a cardiologist and a cardiac surgeon. The baseline operative risk of patients was assessed using the logistic European System for Cardiac Operative Risk Evaluation (EuroSCORE II) and the presence of comorbidities. Procedural success was defined as correct implantation and normal function of the aortic prosthesis in the absence of periprocedural death. Mortality, myocardial infarction (MI), stroke, and vascular complications were defined according to the Valve Academic Research Consortium II (VARC-2) definitions [4]. We also considered the endpoint of hospitalization due to symptoms of cardiac or valvular decompensation or hospitalization for cardiovascular reasons within 30 days after the procedure. A permanent pacemaker was implanted if advanced atrioventricular (AV) block developed, in accordance with the European Society of Cardiology guidelines for patients with acquired AV block in special situations.

### 2.5. Follow-Up

Clinical outcomes and adverse events documented at specific time intervals—30 days, 6 months, and 12 months—were meticulously compiled. All patients were contacted via phone and followed up with through the electronic medical portal eSveikata.lt.

### 2.6. Statistical Analysis

Statistical analysis was performed using SPSS version 27.0 software (IBM Corp., Armonk, NY, USA). The Shapiro–Wilk test and estimated skewness coefficients were used to test hypotheses regarding the normal distribution of the observed interval variables. Data are presented as mean ± SD values when normally distributed and median values (25–75%) when not. The Mann–Whitney test was used to compare the interval characteristics of the two groups, due to unequal group sizes. Differences in symptom frequency were assessed via Chi-square test with Fisher’s correction. Factors potentially influencing death from cardiac causes were identified using backward stepwise binary multivariate logistic regression. Factors that significantly differed between the groups of surviving and deceased patients and the age groups were included in the initial logistic regression model. Typical statistical hypothesis significance levels applied, where *p* < 0.05 indicates statistical significance and *p* > 0.05 indicates statistical insignificance.

## 3. Results

The study included 224 patients. The mean age of patients in the non-death group was 80 ± 6.17 years (range: 49–91), while in the cardiac death group, it was 81.5 ± 6.14 years (range: 70–94). The differences were not statistically significant (*p* = 0.079). Gender differences between the groups were also not statistically significant, with 37.7% males and 62.3% females in the non-death group compared to 50% males and 50% females in the cardiac death group (*p* = 0.304). A history of myocardial infarction was more common in the cardiac death group (38.9% vs. 20.6%; *p* = 0.073). Most patients in both groups were classified as NYHA class III or IV (*p* = 0.264) (Table 1).

During this study, 199 (88.8%) patients survived the transcatheter aortic valve implantation (TAVI) procedure, while 25 (11.2%) died. Among the deceased, 18 (8.0%) deaths were for cardiac reasons. Deaths from cardiac causes occurred at various periods post-procedure: 4 (22.2%) during procedure, 4 (22.2%) within 1 month, 7 (38.9%) within 6 months, and 3 (16.7%) within 12 months (Table 2). A total of 20 patients were hospitalized for cardiovascular indications. Four patients had myocardial infarction (Table 3). An increase in hospitalization frequency was observed: 27.8% of study patients who died due to cardiac reasons were hospitalized compared to 7.5% of survivors (*p*-value > 0.05). No significant differences were detected in the frequency of strokes and myocardial infarctions between the two groups. However, medical and procedural characteristics associated with the risk of death due to cardiac reasons were identified. Specifically, the presence of calcification in the non-coronary cusp was significantly higher among survivors (91.3%) compared to those who died due to cardiac reasons (68.8%), with a *p*-value of 0.005. Among the outcomes, significant differences in mitral valve leakage before TAVI and AoV velocity change were also observed: in the group of deceased patients, greater valve leakage (*p* = 0.015) and lower AoV velocity change (*p* = 0.031) were recorded more often (see Table 4). The fluoroscopy time and contrast volume were significantly higher in patients who died due to cardiac reasons (with *p*-values of <0.001 and 0.005, respectively), suggesting a possible link between the duration of the procedure/amount of contrast used and the risk of death from cardiac reasons, as presented in Table 4, in addition to binary logistic regression models for predicting cardiac death. Each additional year of age increases the likelihood of the outcome by 16% (OR = 1.16, 95% CI from 1.03 to 1.31, *p* = 0.012), indicating a consistent effect across the range. For patients with a left ventricular ejection fraction (LVEF) less than 50%, the odds of the outcome were 3.49 times higher than for patients with an LVEF ≥ 50%, although this result was on the threshold of statistical significance (OR = 3.49, 95% CI from 0.98 to 12.45, *p* = 0.054). Therefore, the risk factors for mortality potentially include an LVEF less than 50%. The presence of calcification in the non-coronary cusp was associated with significantly lower odds of the outcome (OR = 0.21, 95% CI from 0.05 to 0.91, *p* = 0.037). Each additional minute of fluoroscopy time increased the odds of the result by 8% (OR = 1.08, 95% CI from 1.03 to 1.13, *p* = 0.002), indicating a significant association. For each additional unit of contrast volume used, the odds of the outcome increased by 1% (OR = 1.01, 95% CI from 1.00 to 1.02, *p* = 0.047), which was also statistically significant. A higher value, indicating a greater decrease in aortic valve velocity (AoV) post-TAVI, was associated with lower odds of the outcome (OR = 0.34, 95% CI from 0.13 to 0.85, *p* = 0.021), suggesting that a lesser improvement in AoV after TAVI may be considered as a risk factor for mortality.

## 4. Discussion

This study analyzed the demographic and clinical characteristics of patients who underwent transcatheter aortic valve implantation (TAVI) and identified differences between those who survived and those who experienced cardiac-related mortality. The findings provide insights into the potential factors influencing the observed outcomes, reinforcing the importance of conducting a comprehensive patient assessment before the procedure [1].

The median age in the non-death group was 80 ± 6.17 years (range, 49–91), while that in the cardiac death group was 81.5 ± 6.14 years (range, 70–94). Although the cardiac death group was slightly older, the difference did not reach statistical significance (*p* = 0.079). Advanced age is widely recognized as a significant predictor of adverse outcomes in TAVI patients due to reduced physiological reserves [5]. Our findings align with previous studies demonstrating that age is an important—but not necessarily independent—risk factor when other variables are accounted for [3].

The gender distribution between the groups also showed no statistically significant differences (*p* = 0.304). In the non-death group, 37.7% were male and 62.3% were female, compared to an equal distribution of 50% male and 50% female in the cardiac death group. While another authors have suggested that gender may influence outcomes due to anatomical or physiological differences, such effects were not observed in this cohort [2].

History of MI was more common in the cardiac death group (38.9%), when compared to the non-death group (20.6%), although this difference did not reach statistical significance (*p* = 0.073). This trend is consistent with prior research indicating that a history of MI is associated with increased procedural risks and worse post-TAVI outcomes [7]. The hemodynamic burden from pre-existing myocardial damage likely contributes to this association, emphasizing the importance of pre-operatively evaluating cardiac function [8].

Fluoroscopy time has emerged as a novel predictor of outcomes in TAVI patients, as highlighted in recent studies. Longer fluoroscopy durations are associated with an increased risk of complications, including cardiac mortality. Similarly, our findings revealed that prolonged fluoroscopy time (*p* < 0.001) and a higher contrast volume (*p* = 0.005) were significantly associated with adverse outcomes [12]. Other research has corroborated these observations, pointing out the additive risks posed by extended fluoroscopy and high contrast usage, particularly in patients with pre-existing renal dysfunction [13].

The presence of mitral valve regurgitation (MR) was associated with increased cardiac mortality. The interaction of MR with aortic stenosis can exacerbate hemodynamic burdens, and patients with MR often face compounded procedural risks. Addressing MR—either medically or through surgical intervention—is vital for improving outcomes in these high-risk cohorts [7]. Additionally, the calcification patterns of the non-coronary cusp significantly influenced survival, consistent with findings in prior studies. These calcifications—while enhancing valve stability in some cases—can increase procedural complexity when asymmetrically distributed [2,3].

Age and left ventricular ejection fraction (LVEF) emerged as critical determinants of outcomes [5,10]. As age increases, physiological reserves diminish, raising susceptibility to complications. Each additional year of age was found to increase the risk of mortality by 16%, consistent with earlier findings [5]. Reduced LVEF remains a prominent risk factor, emphasizing the importance of comprehensive cardiac assessments before TAVI to identify and manage high-risk patients [10].

Chronic kidney disease and procedural complexity have been identified as independent predictors of poorer outcomes, advocating for the use of advanced imaging techniques and meticulous planning to mitigate these risks [13]. Reducing contrast use during TAVI procedures has been proposed as a means to decrease the incidence of contrast-induced acute kidney injury (CIAKI)—a complication significantly affecting morbidity and mortality [14].

Notably, atrial fibrillation has been identified as a significant predictor of post-TAVI mortality, further underscoring the importance of rhythm management strategies [15]. Meanwhile, frailty indices are valuable tools for pre-operative risk stratification, enabling better patient selection [16].

Advancements in valve design and procedural methods have been instrumental in improving outcomes. Minimalist approaches—including conscious sedation and single-arterial access—have shown promise in reducing complications and recovery times [17,18]. These innovations align with recent findings highlighting the benefits of streamlined TAVI protocols [9]. Additionally, the utility of advanced imaging techniques for enhancing valve positioning and reducing procedural complications has been emphasized [19].

## 5. Limitations

This investigation into the factors that influence the success of TAVI—with a particular focus on fluoroscopy duration and the amount of contrast used—offers important insights for improving patient outcomes. Nevertheless, it is important to recognize a few key limitations. The research was carried out at the Lithuanian University of Health Sciences, meaning that its findings might be specific to the patient population, procedural approaches, and healthcare practices unique to this single institution. Such specificity may limit how applicable the results are across different environments or demographic groups. While the study cohort consisted of 224 participants, this figure might not be sufficient to uncover less-obvious correlations or effects, particularly considering analyses segmented into various patient subgroups. Expanding the sample size could lead to stronger, more conclusive findings and enable a more nuanced examination of the different patient categories.

## 6. Conclusions

Factors including calcification of the non-coronary cusp, mitral valve regurgitation before TAVI, fluoroscopy time, and the volume of contrast used were found to be significantly associated with an increased risk of cardiac death. Future studies with larger, more diverse cohorts are necessary to validate our results and explore the nuanced effects of these and other procedural variables on patient outcomes.

## Figures and Tables

**Table 1:** Baseline clinical, laboratory, echocardiographic, and CT scan characteristics in patient groups.

Variables	Non Death	Cardiac Death	*p*-Value
Age, yrs., median (min–max)	80 (49–91)	81.50 (70–94)	0.079
Gender, *n* (%):			0.304
Male	75 (37.7)	9 (50.0)	
Female	124 (62.3)	9 (50.0)	
BSA m^2^, median (min–max)	1.87 (1.42–2.73)	1.73 (0.55–2.30)	0.026
Arterial hypertension, n (%)	187 (94.0)	17 (94.4)	1.000
Diabetes mellitus, n (%)	54 (27.1)	2 (11.1)	0.168
Coronary artery disease, n (%):			0.205
Yes	73 (36.7)	7 (38.9)	
Yes, after PCI	106 (53.3)	7 (38.9)	
CABG history, n (%)	20 (10.1)	2 (11.1)	0.701
History of myocardial infarction, n (%)	41 (20.6)	7 (38.9)	0.073
Stable angina class, n (%):			0.642
I	1 (0.5)	0 (0.0)	
I–II	72 (36.2)	5 (27.8)	
III	126 (63.3)	13 (72.2)	
Kidney function, n (%):			1.000
Normal	7 (3.5)	0 (0.0)	
Mild or moderate	180 (90.5)	17 (94.4)	
Significant	12 (6.0)	1 (5.6)	
NYHA class, n (%):			0.264
II	58 (29.1)	4 (22.2)	
III	139 (69.8)	13 (72.2)	
IV	2 (1.0)	1 (5.6)	
EuroScore II percent, median (min–max)	3.63 (0.91–34.96)	4.77 (1.68–21.70)	0.107
LVEF, n (%):			0.040
<50%	63 (31.7)	10 (55.6)	
≥50%	136 (68.3)	8 (44.4)	
AoS, n (%):			0.771
Significant	158 (79.4)	14 (77.8)	
Critical	41 (20.6)	4 (22.2)	
AoV regurgitation before TAVI II-III grade, n (%):	83 (37.7)	7 (38.9)	0.752
Bicuspid AoV, n (%)	13 (6.5)	2 (11.1)	0.359
Mitral valve regurgitation before TAVI, n (%):			0.015
None	3 (1.5)	1 (5.6)	
Grade 1	69 (34.7)	2 (11.1)	
Grade 2	109 (54.8)	10 (55.6)	
Grade 3	18 (9.0)	5 (27.8)	
LV relative wall thickness, mm, mean ± SD	0.50 ± 0.10	0.52 ± 0.11	0.404
AoV velocity, m/s, mean ± SD	4.44 ± 0.68	4.13 ± 0.75	0.094
Mean gradient across AoV, mmHg, mean ± SD	48.74 ± 16.34	42.58 ± 15.64	0.130
AoV area, cm^2^, mean ± SD	0.78 ± 0.19	0.76 ± 0.17	0.641
AoV index, mean ± SD	0.41 ± 0.10	0.43 ± 0.08	0.475
AoV diameter, mm, mean ± SD	25.17 ± 2.35	25.21 ± 2.65	0.997
AoV perimeter, mm, mean ± SD	79.54 ± 7.30	80.10 ± 8.36	0.744
AoV diameter, perimeter-derived, mm, mean ± SD	25.31 ± 2.33	25.41 ± 2.70	0.826
AoV annulus diameter according to area, mm, mean ± SD	24.92 ± 2.31	25.00 ± 2.67	0.948
Angle aorto annulus, mean ± SD	51.26 ± 8.67	53.94 ± 12.88	0.594
Calcified left coronary cusp, n (%)	143 (72.6)	11 (68.8)	0.741
Calcified right coronary cusp, n (%)	155 (78.7)	10 (62.5)	0.208
Calcified non-coronary cusp, n (%)	178 (91.3)	11 (68.8)	0.005
AoV calcified volume, n (%):			0.310
Mild	54 (27.4)	7 (43.8)	
Moderate	81 (41.1)	4 (25.0)	
Severe	62 (31.5)	5 (31.3)	
Pulse pressure, mmHg, mean ± SD	59.92 ± 16.82	52.17 ± 15.11	0.057
Contrast volume, ml, median (min–max)	150 (50–400)	175 (100–400)	0.005
AoV velocity after TAVI, m/s, median (min–max)	1.90 (1.10–3.65)	1.99 (1.0–3.20)	0.960
ΔAoV velocity, m/s, mean ± SD	2.43 ± 0.68	2.04 ± 0.75	0.031

Data are presented as mean and SD, standard deviation (minimum–maximum), with or without ranges, or numbers and percentages. Abbreviations: BSA—body surface area; MI—myocardial infarction; PCI—percutaneous coronary intervention; CABG—coronary artery bypass graft; LVEF—left ventricular ejection fraction; NYHA—New York Heart Association; AoS—aortic stenosis; AoV—aortic valve; LV—left ventricular; *p*—significance level.

**Table 2:** Distribution of mortality and cardiac death over time after TAVI.

Non-Death	199 (88.8%)
Overall mortality	25 (11.2%)
Cardiac death	18 (8.0%)
During the procedure	4 (22.2%)
1-month follow-up	4 (22.2%)
6-month follow-up	7 (38.9%)
12-month follow-up	3 (16.7%)

**Table 3:** Comparison of clinical events between non-cardiac death and cardiac death groups.

Events	Undead	Cardio Dead	χ^2^	*p*
	n = 199	n = 18		
Stroke, n (%)	8 (4.0%)	1 (5.6%)	0.098	0.548
Hospitalization, n (%)	15 (7.5%)	5 (27.8%)	8.082	0.016
MI, n (%)	4 (2.0%)	0 (0.0%)	0.369	1.000

Variables: MI, myocardial infarction.

**Table 4:** Relative risk factors for cardiac death after TAVI—binary logistic regression model (0—not dead/1—dead).

Variables	*p*-Value	OR	95% C.I. for OR	
			Lower	Upper
Age	0.012	1.16	1.03	1.31
Pulse pressure before TAVI	0.102	0.97	0.93	1.01
LVEF < 50%	0.054	3.49	0.98	12.45
Calcified non coronary	0.037	0.21	0.05	0.91
Fluoroscopy time	0.002	1.08	1.03	1.13
Contrast volume	0.047	1.01	1.00	1.02
ΔAoV	0.021	0.34	0.13	0.85

Variables: age, pulse pressure before, LVEF < 50% (0, No; 1, Yes), calcified non-coronary (0, No; 1, Yes), fluoroscopy time, contrast volume, ΔAoV (interval variable: higher value means greater reduction after TAVI).

## Data Availability

The data supporting this study are not publicly available due to ethical restrictions. Access to the data may be granted upon reasonable request and subject to approval by the local ethics committee.

## Abbreviations

The following abbreviations are used in this manuscript:

AS	aortic stenosis
AoV	aortic valve
AV	atrioventricular
BSA	body surface area
BEVs	balloon-expandable valves
CABG	coronary artery bypass graft
CIAKI	contrast-induced acute kidney injury
CT	computed tomography
CVDs	cardiovascular diseases
EuroSCORE II	European System for Cardiac Operative Risk Evaluation
LVEF	left ventricular ejection fraction
MI	myocardial infarction
MR	mitral valve regurgitation
NYHA	New York Heart Association
PCI	percutaneous coronary intervention
SAVR	surgical aortic valve replacement
SEV	self-expandable valve
SPSS	statistical analysis was performed using SPSS
TAVI	transcatheter aortic valve implantation
VARC-2	Valve Academic Research Consortium II
